# Studies of Electrical Parameters and Thermal Stability of HiPIMS Hafnium Oxynitride (HfO_x_N_y_) Thin Films

**DOI:** 10.3390/ma16062539

**Published:** 2023-03-22

**Authors:** Mirosław Puźniak, Wojciech Gajewski, Aleksandra Seweryn, Marcin T. Klepka, Bartłomiej S. Witkowski, Marek Godlewski, Robert Mroczyński

**Affiliations:** 1 Warsaw University of Technology, Institute of Microelectronics and Optoelectronics, Koszykowa 75, 00-662 Warsaw, Poland; 2 TRUMPF Huettinger, Marecka 47, 05-220 Zielonka, Poland; 3 Institute of Physics, Polish Academy of Sciences, Aleja Lotnikow 32/46, 02-668 Warsaw, Poland

**Keywords:** HfO_x_N_y_, MOS, HiPIMS, reactive magnetron sputtering, thermal stability, electrical parameters, structural characterization

## Abstract

This work demonstrated the optimization of HiPIMS reactive magnetron sputtering of hafnium oxynitride (HfO_x_N_y_) thin films. During the optimization procedure, employing Taguchi orthogonal tables, the parameters of examined dielectric films were explored, utilizing optical methods (spectroscopic ellipsometry and refractometry), electrical characterization (C-V, I-V measurements of MOS structures), and structural investigation (AFM, XRD, XPS). The thermal stability of fabricated HfO_x_N_y_ layers, up to 800 °C, was also investigated. The presented results demonstrated the correctness of the optimization methodology. The results also demonstrated the significant stability of hafnia-based layers at up to 800 °C. No electrical parameters or surface morphology deteriorations were demonstrated. The structural analysis revealed comparable electrical properties and significantly greater immunity to high-temperature treatment in HfO_x_N_y_ layers formed using HiPIMS, as compared to those formed using the standard pulsed magnetron sputtering technique. The results presented in this study confirmed that the investigated hafnium oxynitride films, fabricated through the HiPIMS process, could potentially be used as a thermally-stable gate dielectric in self-aligned MOS structures and devices.

## 1. Introduction

Several high-k materials, including metal oxides [1], nanolaminates [2], and silicates [3], were intensively studied for their potential applications in high-performance or low-power complementary metal-oxide semiconductor (CMOS) devices. The commonly studied hafnium oxide (HfO_2_) has proven wide applications [4,5,6], a relatively high permittivity value [7], a large band-gap [8], reasonably good band offset in contact with silicon substrate [9], and compatibility with polysilicon, as well as metal gate electrodes [10]. However, the crystallization temperature of HfO_2_ is relatively low, leading to the growth of grain boundaries—which are perfect paths for oxygen, boron, and other impurities to penetrate into the semiconductor/dielectric interface [11]. All these effects result in gate dielectric long-term reliability degradation, which must be reduced to maintain reasonably high device performance.

One possible solution to improve the insulating properties and increase the crystallization temperature is nitrogen incorporation into the Hf-based high-k layer to form hafnium oxynitride (HfO_x_N_y_) [12]. It was reported that nitrogen introduction—even in small amounts—in dielectric layer bulk (up to 5 at%) led to increases of about 300 °C in crystallization temperature, larger permittivity levels, lower leakage current densities with similar equivalent oxide thickness values (*EOT*), and better insulating properties, as compared to stochiometric hafnium oxide (HfO_2_) films [13]. Numerous techniques for the fabrication of hafnium oxynitride films can be used, i.e., high-temperature reoxidation of physically vapor-deposited hafnium nitride (HfN) to form HfO_x_N_y_ [14], chemical vapor deposition (CVD) [15] or metalorganic chemical vapor deposition (MOCVD) [16]. Still, the reactive magnetron sputtering process remains a desirable method of fabrication. The material synthesis offers many advantages, such as low-temperature processing (i.e., at room temperature), good uniformity of the deposited material, reasonable control over stoichiometry, and quality of the sputtering parameters [17]. A pulsed magnetron sputtering (PMS) mode is commonly used to fabricate hafnium oxynitride films; however, high-power impulse magnetron sputtering (HiPIMS) offers many advantages over the standard PMS mode. A HiPIMS process can be characterized by a physical vapor deposition (PVD) technique that utilizes highly-energetic pulses at a low-duty cycle, applied to a conventional PMS magnetron sputtering process. HiPIMS can be characterized by highly ionized fluxes of film-forming particles and enhanced energies of the ions bombarding the growing films, resulting in structural changes and densification without a substrate bias or more effortless scalability to an industrial level [18,19,20,21]. However, experimental studies of HiPIMS materials and their potential applications in gate dielectric layers for MOS structures and devices are notably lacking.

In this work, the fabrication and optimization of the technology of HfO_x_N_y_, deposited employing the HiPIMS method, were demonstrated. The experimental runs were prepared using the Design of Experiments (DoE) approach, namely, Taguchi orthogonal tables [22]. The technique allowed for the effective reduction of several experiments to investigate the dependencies between variables of process parameters, i.e., magnetron sputtering and output parameters, i.e., the parameters of the examined material or structure [23]. As an effect of the optimization procedure, the set of variables of the magnetron sputtering process was designed to obtain the HfO_x_N_y_ film with the potential best electrical properties. The changes in the parameters of investigated films during the optimization procedure were observed through optical methods and the electrical characterization of purposely fabricated MOS structures. The structural parameters and surface morphologies of dielectric films were investigated using atomic force microscopy (AFM), grazing-angle incidence X-ray diffraction (GIXRD), and X-ray photoelectron spectroscopy (XPS). Finally, the electrical performance and thermal stability values of HfO_x_N_y_ films fabricated using HiPIMS and typical pulsed magnetron sputtering methods were compared. The findings presented in this work showed the fabricated HiPIMS films to be promising candidates in electronic applications, e.g., as the gate dielectric material in MOS structures and devices.

## 2. Materials and Methods

### 2.1. Fabrication and Optimization of Hafnium Oxynitride Thin Films

The HfO_x_N_y_ films were formed on (100) p-type silicon (Si) substrates with a resistivity of 1 ÷ 10 Ωcm, and quartz substrates, employing a reactive magnetron sputtering process. Before the fabrication of dielectric films, Si substrates were cleaned utilizing the modified Radio Corporation of America (RCA) method, assuming a procedure using the following solutions: (1) H_2_O_2_:H_2_SO_4_ = 1:2 for 5 min; (2) H_2_O:NH_4_OH:H_2_O_2_ = 5:1:1 at 80 °C for 10 min; (3) H_2_O:HCl:H_2_O_2_ = 6:1:1 at 80 °C for 10 min; and (4) HF:H_2_O = 1:50 for 5 min, with deionized (DI) water intensive rinsing between each of the cleaning steps. The quartz substrates were cleaned ultrasonically by rinsing them in acetone and ethanol, followed by a deionized water bath and nitrogen drying. A commercial PlasmaLab System 400 (Oxford Instruments Plasma Technology, Bristol, UK) was used with a TruPlasma Highpulse 4000 G2 (Trumpf Huettinger, Zielonka, Poland) generator to activate the glow discharge in the HiPIMS mode, while the reference dielectric layers were obtained through the PMS mode with the Pinnacle Plus (Advanced Energy, Littlehampton, UK) generator. The substrates were positioned on-axis with respect to the sputtering beam, and the target-to-substrate distance was 20 cm.

The experiment was composed of two stages. The first stage aimed to investigate the influence of the sputtering parameters on the electrical properties of HfO_x_N_y_ films and design a set of parameters to obtain, potentially, a high-quality dielectric material. To that end, MOS capacitors with hafnium oxynitride films as the gate dielectric material were formed. The technological runs were designed according to the orthogonal tables. The L_9_(3)^4^ Taguchi table was utilized in this work. The influence of power applied to the electrodes, the pressure in the reactive chamber, and the argon and nitrogen flow, each with three variables, were investigated on the selected output properties. There were nine total processing steps. The processing time in each step was kept constant at 5 min. Table 1 presents the values of process parameters for optimizing the electrical properties of HfO_x_N_y_ films.

The obtained results allowed for the anticipation and design of a set of process parameters to fabricate dielectric layers with high electrical performance parameters. In the second stage of the experimental part, the verification of the optimization procedure was presented. The two sets of HiPIMS processes were designed to obtain dielectric materials with (1) good electrical parameters (i.e., optimal process) and (2) intentionally worse electrical parameters (i.e., non-optimal process). This procedure aimed to show that the DoE method was performed correctly and the fabrication of dielectric layers was controllable. In this step, process time was fitted to fabricate a dielectric film with a thickness of ~30 nm. MOS structures were fabricated using the selected HiPIMS processes of HfO_x_N_y_. Split experiments, with annealing of fabricated structures, were implemented using a standard furnace at 300 °C and 800 °C in an argon atmosphere for 30 min. After annealing, the PMS process was used to fabricate aluminum (Al) as the top-metal gate. Standard photolithography in ultraviolet (UV) radiation was used to structure the Al contact pads. Finally, the MOS structures were divided as-grown, without any annealing treatment, and annealed at either 300 °C or 800 °C.

### 2.2. Electrical Characterization of MOS Devices

Electrical measurements of the fabricated structures were performed using the Keithley 4200 semiconductor characterization system (Tektronix, Beaverton, OR, USA) equipped with SUSS PM-8 probe station (SUSS MicroTec Semiconductor, Garching, Germany), with the probe-shield option allowing low-noise characterization of fabricated devices. MOS capacitors, with a gate area of A = 1.8 × 10^−4^ cm^2^, were measured, allowing the determination of the electrical parameters of the examined stacks. Possible changes in the electrical properties were monitored through capacitance-voltage (C-V) and current-voltage (I-V) measurements. The basic electrical parameters of obtained MOS devices were evaluated as presented in [24]. The equivalent oxide thickness (*EOT*) of the dielectric stacks investigated in this work were estimated based on the maximum capacitance (*C_MAX_*) of the MOS structure in the accumulation regime. We used the following equation:

(1) $$EOT=\frac{\varepsilon_{0}\cdot \varepsilon_{i}\cdot A}{C_{MAX}}\;\left(\mathrm{nm}\right)$$

where *ε*_0_ is vacuum permittivity, *ε_i_* is electric permittivity of the dielectric film, and *C_MAX_* is obtained using the two-point method of Majkusiak and Jakubowski [25]. The latter approach considers a possible leakage current in the accumulation regime, thus, it is frequently used to extract a maximum capacitance value. However, similar values of *C_MAX_* were determined after obtaining specific values directly from the C-V characteristic:

(2) $$C_{MAX}=\left(\frac{C_{1}+C_{2}}{2}+\frac{kT}{q}\left|\frac{C_{2}-C_{1}}{U_{G2}-U_{G1}}\right|\right)+\sqrt{{\left(\frac{C_{1}+C_{2}}{2}+\frac{kT}{q}\left|\frac{C_{2}-C_{1}}{U_{G2}-U_{G1}}\right|\right)}^{2}-C_{1}C_{2}}\;\left(F\right)$$

where *A* is a gate area, *kT/q* = 0.0258 V, and *C*_1_, *C*_2_, *U_G_*_1_, and *U_G_*_2_ are coordinates of two points selected in the accumulation regime of C-V characteristic of MOS structure.

The electric permittivity of hafnium oxynitride films was calculated using the following equation:

(3) $$\varepsilon_{i}=C_{MAX}\frac{t_{i}}{A}\;\left(F\cdot {\mathrm{cm}}^{-1}\right)$$

where *t_i_* is the thickness of HfO_x_N_y_ measured after spectroscopic ellipsometry. For this purpose, the spectroscopic ellipsometer UVISEL (Horiba Jobin-Yvon, Lille, France) was used. The apparatus allowed for measurements in the wavelength range of 190–850 nm. For the modeling and fitting of the ellipsometric data, the Tauc–Lorentz (TL) dispersive model was used. All samples were measured at 70° angle of incidence.

For flat-band voltage value (*V_fb_*) estimation, at first, the flat-band capacitance was calculated, using the following equation:

(4) $$C_{fb}=\frac{C_{i}\cdot \left(\frac{\varepsilon_{s}\cdot A}{\lambda }\right)}{C_{i}+\left(\frac{\varepsilon_{s}\cdot A}{\lambda }\right)}\;\left(F\right)$$

where *C_fb_* is a flat-band capacitance, *C_i_* is a dielectric stack capacitance, *ε_s_* is the permittivity of Si, *A* is a gate area, and *λ* is Debye length, calculated as follows:

(5) $$\lambda ={\left(\frac{\varepsilon_{s}\cdot kT}{q^{2}\cdot N_{s}}\right)}^{\frac{1}{2}}\;\left(\mathrm{cm}\right)$$

where *q* is an electron charge and *N_s_* is the dopant concentration. Based on the above evaluation, we were able to find the flat-band voltage value of the MOS structure directly from a particular C-V curve.

The effective charge density (*Q_eff_/q*) of the dielectric stack was evaluated, as follows:

(6) $$\frac{Q_{eff}}{q}=\frac{\varepsilon_{i}}{t_{i}}\cdot \left(\varphi_{MS}-V_{fb}\right)\left({\mathrm{cm}}^{-2}\right)$$

where, for an aluminum metal gate, the work function (*φ_MS_*) was assumed as:

(7) $$\varphi_{MS}=\varphi_{Al}-\left[\varphi_{Si}+\frac{E_{g}}{2}-\varphi_{F}\right]≅-0.6-\varphi_{F}\;\left(\mathrm{eV}\right)$$

where *φ_F_* is the Fermi potential. The average interface state density in the middle of the forbidden silicon band (*D_itmb_*) was estimated using the Terman method [26]. The electrical measurements were done using around 30 test MOS capacitors. All presented characteristics in the manuscript were representative.

### 2.3. Structural Characterization of Dielectric Films

The structures and chemical compositions of the examined samples were studied employing spectroscopic ellipsometry, reflectometry, atomic force microscopy (AFM), grazing-angle incidence X-ray diffraction (GIXRD), and X-ray photoelectron spectroscopy (XPS). The thickness and optical properties of the investigated hafnium oxynitride thin-films were determined using a Jobin-Yvon UVISEL (Horiba, Lille, France) spectroscopic ellipsometer in a wavelength range from 190 nm to 850 nm. An FR-pRo reflectometer (Thetametrisis, Athens, Greece) was employed to analyze thin-film reflectance/transmittance characteristics. Grazing incidence X-ray diffraction data were collected with a Panalytical X’Pert Pro MRD diffractometer (Panalytical, Almelo, The Netherlands). The diffractometer was equipped with an X-ray tube, generating radiation at a wavelength of 1.54056 Å, and a hybrid two-bounce Ge (220) monochromator with a Pixel detector [27]. Data were gathered in the 2theta range 10–80°, in steps of 0.039°, with an adequate scan time of 1000 s per step. The incidence angle was set at ω = 0.54°. Those parameters were chosen as the maximum intensity of the signal from the HfO_x_N_y_, and the minimum signal from the semiconductor substrate was observed. XPS measurements were performed on system from Prevac (Prevac, Rogow, Poland) with a Scienta R4000 (Scienta Omicron, Uppsala, Sweden) hemispherical analyzer (pass energy 200 eV) and monochromatic Al K_α_ (1486.7 eV) excitation, working with a power of 150 W. The full width at half maximum (FWHM) of the 4f7/2 Au line, measured under the same experimental conditions, was 1.1 eV. The energy scale was calibrated, setting the C1s line at 285.0 eV. Samples were measured as received. The spectra were analyzed using the commercial CASA XPS software package (Casa Software Ltd., Teignmouth, UK, version 2.3.25PR1.0) with the Shirley background. The spectra were fitted with a mixed Gaussian–Lorentzian GL (30) function, as reported in [27]. The surface morphology was investigated using AFM (Bruker Dimension Icon, Santa Barbara, CA, USA), using PeakForce Tapping (Bruker, Billerica, MA, USA) and silicon nitride probes with sharp tips (tip radius—2 nm). The surface roughness was determined by a root mean square (*RMS*) roughness of the AFM height measurements, using images taken from a 10 × 10 μm^2^ region. The surface morphology of the examined dielectric layers was measured using Si substrates.

## 3. Results and Discussion

**Figure 1:**
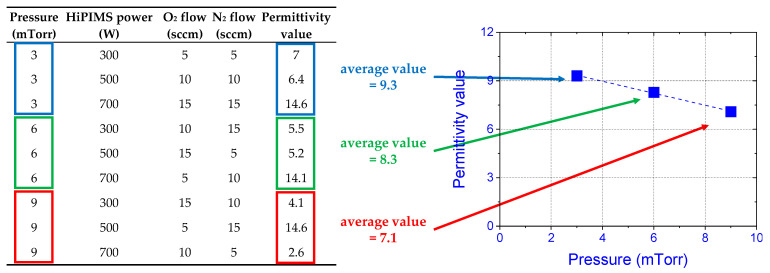
Graphing methodology used in orthogonal tables, employed during the analysis of dependencies between process and material parameters; the example shows the trend (dashed line) of the influence of pressure in the reactive chamber on the permittivity value of HfO_x_N_y_ films (see Figure 2).

**Figure 2:**
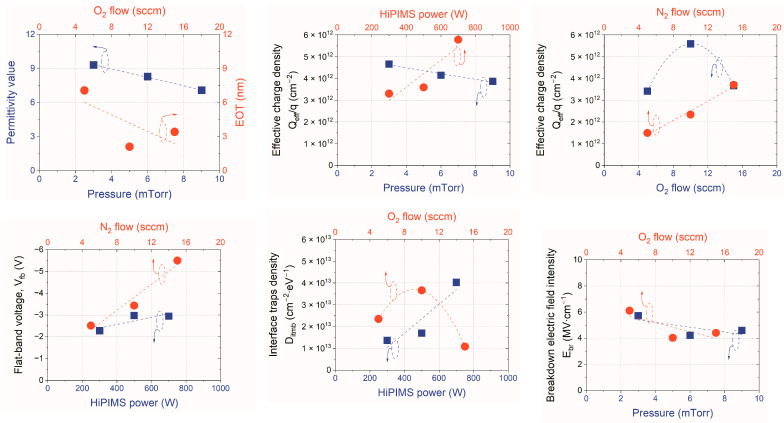
Most essential trends (dashed lines) obtained according to the Taguchi approach employed in this study, which are taken into account to set the ultimate process parameters for fabrication of HfO_x_N_y_ layers.

Applying the DoE method and implementing Taguchi orthogonal tables allowed for the effective evaluation of the obtained results [28,29]. The impacts of specific input (process) parameters on investigated output (material) parameters could be analyzed by particular plots, showing trends of dependencies between input and output parameters. Figure 1 illustrates the methodology of graphing in the Taguchi approach, while Figure 2 depicts the most critical dependencies between process parameters and electrical parameters of the obtained hafnium oxynitride thin films that were used during the investigations performed in this study.

The graphing methodology in the Taguchi approach is based on the analysis of output values of investigated parameters. For example, Figure 1 shows how the trend of the dependence of the influence of pressure on the permittivity of the examined hafnium oxynitride films was plotted. Each point of the trend resulted from averaging the obtained values in a set of processes in which the value of a particular input parameter was constant. This work used the same methodology during investigations of all dependencies and optimization procedures presented in the text below. The crucial dependencies for further optimization of electrical parameters of HfO_x_N_y_ films are illustrated in Figure 2. The optimization procedure required the analysis of large numbers of trends and quantities of data. Therefore, from an optimization perspective, in some cases, it was necessary to find a compromise and anticipate the most favorable values of the process parameters. 

The trends presented in Figure 2 proved that increasing oxygen flow decreased the *EOT* value of a dielectric film. Moreover, the lower oxygen flow increased the intensity of the electric field, which caused the breakdown phenomenon; however, the trend was not a strong function of the O_2_ flow. Simultaneously, the peak of the O_2_ flow was observable, which led to high values of effective charge and interface trap densities. Considering all analyzed trends, it was observed that the better electrical parameters of the ultimate HfO_x_N_y_ film should be characterized by material deposited at higher oxygen flow. Thus, 20 sccm of oxygen flow was selected in the next step of the optimization procedure.

As for the influence of the pressure in the reactive chamber, the demonstrated trends pointed out that lower pressure correlated to higher permittivity and higher breakdown electric field intensity. The influence of the pressure on *Q_eff_*/*q* value was a weak function, i.e., as the pressure changed from 3 to 9 mTorr, the effective charge density only decreased by 0.5 × 10^12^ cm^−2^. Thus, in further processing of high-quality hafnium oxynitride films, a lower pressure value, i.e., 2 mTorr, was chosen. Similar analyses were performed while setting the ultimate values of HiPIMS power, applied to the reactive chamber and nitrogen flow. It was observed that decreasing the power during processing resulted in the improvement of all monitored electrical parameters, i.e., the flat-band voltage value (*V_fb_*), effective charge, and interface trap densities (*D_itmb_*). Furthermore, better parameters could be acquired by setting lower N_2_ flow, in the case of nitrogen flow.

The observations drawn from the analysis of the acquired trends were used to design a set of recipes with parameters that allowed the sputtering of dielectric films with a designed electrical performance. Based on conclusions and assumptions derived from the trends depicted in Figure 2, two sets of experimental runs were designed. The first (1) had better electrical parameters of the HfO_x_N_y_ layer, i.e., the first was the optimal process, and the second (2) had intentionally-deteriorated electrical parameters of the HfO_x_N_y_ layer, i.e., the second was the non-optimal process. The values of pressure, HiPIMS power, and O_2_/N_2_ flow selected for each set of parameters are presented in Table 2.

**Figure 3:**
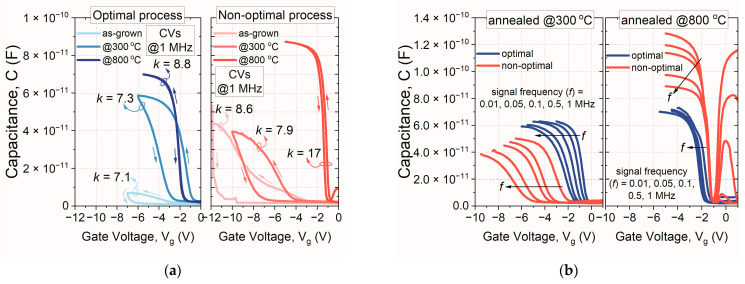
Comparison of the hysteresis loop (**a**) and frequency dispersion (**b**) of C-V characteristics of MOS structures with HfO_x_N_y_ films, fabricated using designed sets of parameters for reactive magnetron sputtering processes, i.e., optimal and non-optimal processes.

In the next stage of the performed study, MOS capacitors with HfO_x_N_y_ films (deposited employing optimal and non-optimal processes) were fabricated. Such a methodology allowed for verification of the obtained trends from the previous optimization stage. The thickness of HfO_x_N_y_ films was set at ~30 nm in all cases. Figure 3 presents a comparison of the hysteresis loops (a) and frequency dispersion (b) of C-V characteristics of MOS structures. The thermal stability of the fabricated films was also examined.

Several conclusions and findings were drawn through analysis of the data presented in Figure 3. First, the annealing procedure used for the HfO_x_N_y_ films resulted in the increase of their permittivity, which was clearly observed in both HiPIMS processes (Figure 3a). However, in the case of the non-optimal process, the annealing at 300 °C caused a slight decrease of *k* value, compared to as-grown hafnium oxynitride film. Maximum permittivity (*k* = 17) was obtained for the HfO_x_N_y_ film fabricated by a non-optimal process and annealed at 800 °C; however, in the case of the optimal approach, the hafnium oxynitride film was characterized by a *k* value of 8.8, as usually found in the literature [30].

The trending increase of *k* value resulted from the rebuilding and passivation of dangling bonds in the dielectric material bulk due to the thermal treatment [31]. Moreover, the hysteresis magnitude, flat-band voltage value (Figure 3a), and frequency dispersion of C-V characteristics (Figure 3b) also decreased due to the thermal treatment. However, all of these parameters were improved for materials fabricated using the optimal HiPIMS process. It should be noted that the annealing process also resulted in the complete disappearance of C-V curves’ frequency dispersions, suggesting that the best electrical parameters were for films annealed at 800 °C. However, the maximum capacitance in the accumulation regime (*C_MAX_*) showed unusual behavior, in the case of HfO_x_N_y_ film formed employing a non-optimal process (Figure 3b). As the signal frequency increased, the *C_MAX_* decreased, which could have been related to the deteriorated series resistance. Such a phenomenon would limit the practical applications of such dielectric materials in semiconductor structures and devices.

**Figure 4:**
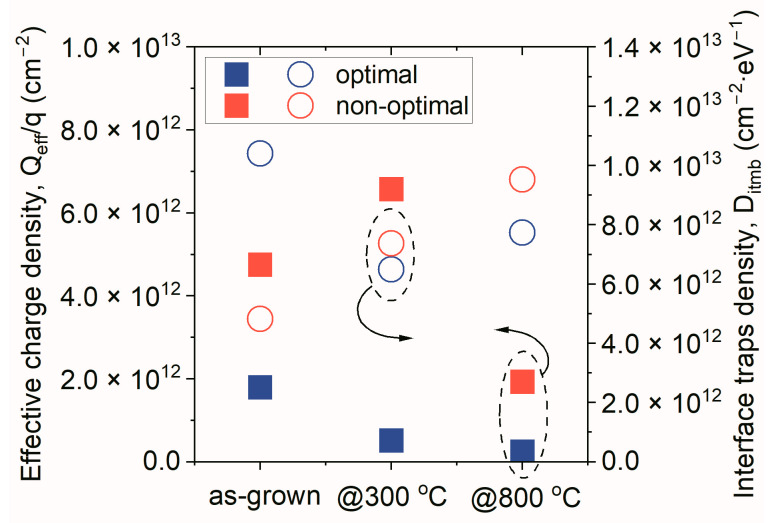
*Q_eff_/q* and *D_itmb_* values, estimated from analysis of C-V characteristics of MOS devices with HfO_x_N_y_, as the gate dielectric film fabricated employs optimal and non-optimal HiPIMS processes.

All findings mentioned above were proven by the results shown in Figure 4, where electrical parameters, evaluated from the C-V characteristics of examined MOS structures, are depicted. It was noted that *Q_eff_/q* and *D_itmb_* values improved with increases in the annealing temperature, for the HfO_x_N_y_ film obtained by the optimal process. On the contrary, in the non-optimal process, only bulk properties of the dielectric film annealed at 800 °C were improved. In contrast, interface properties deteriorated as the annealing temperature increased. It must also be underlined that, in the case of annealed structures, in all cases, the electrical parameters of MOS devices with gate dielectric films formed using the optimal process were improved, as compared to the non-optimal fabrication procedure.

**Figure 5:**
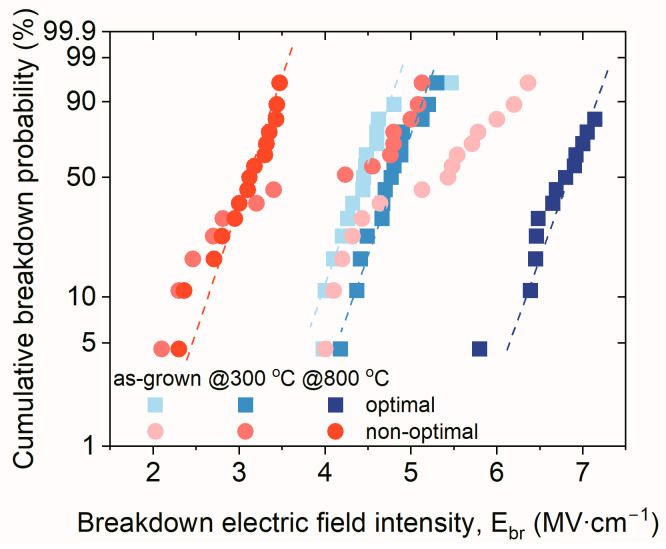
Cumulative breakdown statistics of MOS structures with examined hafnium oxynitride thin films, fabricated employing optimal and non-optimal HiPIMS processes; dashed lines indicate obtained trends.

The measurements of current-voltage characteristics of MOS test structures exposed additional findings that supported the accuracy of the Taguchi approach used for the optimization of the variables of optimal process parameters. In Figure 5, Weibull plots of investigated MOS structures are compared. These plots compared the statistics of the breakdown phenomenon of investigated hafnium oxynitride films in this study. At first, it was worth noting that the distribution of the breakdown electric field intensity was much narrower in the case of structures with as-grown HfO_x_N_y_ films fabricated employing the optimal process, i.e., in the case of ~80%, the breakdown phenomenon occurred in the range 4–4.5 MV/cm.

On the contrary, MOS structures with dielectric films formed using the non-optimal process were characterized by the distribution of *E_br_* values in the range 4–6.5 MV/cm, which indicated a significant non-repeatability of electrical behavior. After the annealing procedure, the insulating properties of the HfO_x_N_y_ films fabricated using the non-optimal process further deteriorated. However, in the case of samples annealed at 800 °C, the distribution of *E_br_* values was very narrow (2.5–3.5 MV/cm for ~80% of examined structures). These results eliminated the obtained dielectric films from potential electronic applications, e.g., as gate dielectric materials. In the optimal process, the breakdown electric field distribution was 6.5–7 MV/cm for 90% of considered MOS structures. This demonstrated the repeatable performance characteristics and superior electrical properties of HiPIMS HfO_x_N_y_ layers. Considering the optimization results, it was concluded that applying the orthogonal tables as in the DoE method allowed for the fabrication of MOS structures with HiPIMS HfO_x_N_y_ layers that had improved electrical properties.

**Figure 6:**
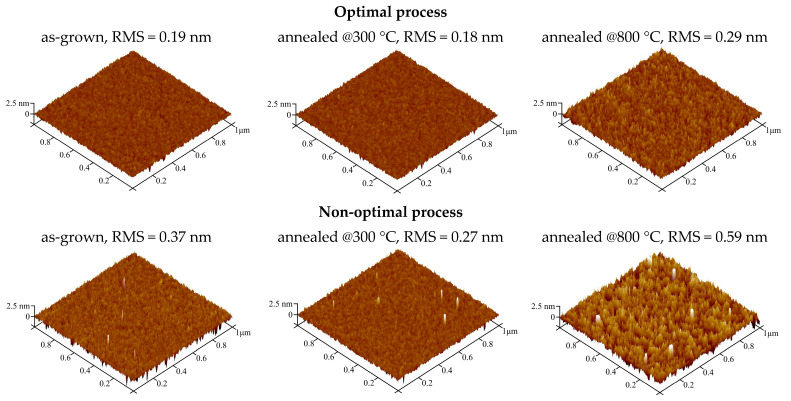
Morphology of HfO_x_N_y_ films characterized by AFM; RMS values of the investigated dielectric layers are also shown.

The performed structural examinations of the fabricated films offer explanations for, and insight into, changes in the electrical behavior of the investigated dielectric layers. Figure 6 compares the topography map, obtained through AFM measurements of the surfaces of hafnium oxynitride thin films fabricated using both optimal and non-optimal processes. The essential differences in the homogeneity of the surfaces of HfO_x_N_y_ films could be observed by employing a specific HiPIMS procedure.

In the case of the optimal strategy, a noticeable increase in the root mean square (*RMS*) factor was observed, i.e., 0.19 nm and 0.29 nm for as-grown dielectric material and material annealed at 800 °C, respectively. In the non-optimal process, the *RMS* factor for all dielectric material significantly increased compared to consecutive HfO_x_N_y_ layers obtained using the optimal method. In the case of dielectric film annealed at 800 °C, the surface of the coating was inhomogeneous and showed a significant amount of crystallinity. Increasing the crystalline phase in high-k dielectric films is a general problem that may lead to generating several leakage paths and, ultimately, to the breakdown of MOS structures. This could explain the deterioration of the electrical behavior discussed above, as well as the results presented in Figure 5. We also observed modification of HfO_x_N_y_ thin films, from an amorphous into a polycrystalline state, after high-temperature treatment (discussed below), as well as a significant increase of leakage current [32].

**Figure 7:**
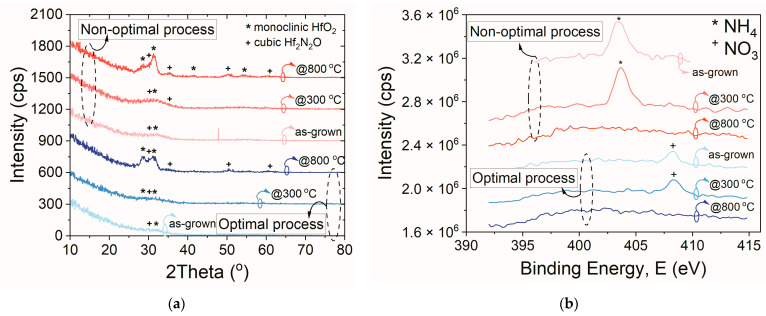
GIXRD patterns (**a**) and N1s photoelectron spectra (**b**) of HfO_x_N_y_ thin films (as-grown and annealed at 300 °C or 800 °C).

Further proof that the deterioration of insulating properties of the investigated HiPIMS hafnium oxynitride films could be correlated with changes in the structural properties is depicted in Figure 7a, where the grazing incidence X-ray diffraction (GIXRD) spectra of the films examined in this work can be found. Regardless of the process type used, the as-grown HfO_x_N_y_ films and those annealed at 300 °C were characterized by a pattern typical for amorphous material. However, a slight increase in the signal intensities was observed at 2theta ~30°, especially for materials deposited using the non-optimal process. However, all signals had low intensities and possibly originated from semiconductor substrates.

After annealing at 800 °C, structural rebuild and increased crystallinity levels in the HfO_x_N_y_ bulk were demonstrated, as shown in Figure 7a. The structural analysis of the collected spectra revealed that the dielectric material was presumably composed of two phases, i.e., a monoclinic crystalline phase, with the chemical composition of stoichiometric HfO_2_, and a cubic phase with the chemical composition of Hf_2_N_2_O. However, due to the low thicknesses of the examined materials, the gathered patterns were characterized by low peak intensities. For this reason, reflexes originating from various phases were able to overlap. Moreover, some of the peaks, with particular degrees of probability, could be qualified to multiple other phases, such as HfO_2_ orthorhombic (space group Pbcm), HfO_2_ cubic (space group Fm-3m), or Hf_2.01_N_2.68_ tetragonal (space group I4/m). The contemporary presence of two or more phases in HfO_2_ and HfO_x_N_y_ films has been widely reported [33]. However, the most important observed conclusion, based on the data analysis shown in Figure 7a, was that the intensities of peaks originating from the monoclinic HfO_2_ phase and cubic Hf_2_N_2_O phase were different for both investigated HfO_x_N_y_ films. In the case of the hafnium oxynitride layer fabricated using the optimal process, both peaks had similar intensities. In contrast, in the case of the non-optimal procedure, the peak intensity originating from the cubic phase was much higher. This finding proved that the superior electrical properties of the investigated HfO_x_N_y_ film correlated to a greater degree of intermixing of both phases. Thus, the excess nitrogen-bound phase—as compared to the oxygen phase in the layer bulk—could cause deteriorations of the electrical properties of hafnium oxynitride films [34].

Further conclusions were drawn after observing the data shown in Figure 7b, where the comparison of photoelectron spectra (N1s) of both layers is presented. A detailed analysis of the nitrogen content in the examined samples was done, based on [35]. A small compound of N was identified in the volume of the HfO_x_N_y_ using standard sensitivity factors, i.e., about 1% and 4% in the case of the optimal and non-optimal processes, respectively. In both as-grown samples and those annealed at 300 °C, a nitrogen component close to the binding energy of the NH_4_ (402.2 eV) and NO_3_ (407.2 eV) groups was identified (for optimal and non-optimal processes, respectively). The thermal treatment resulted in nitrogen disappearance from the dielectric layer surface (see Figure 7b, samples annealed at 800 °C). It could be speculated that the nitrogen fully diffused toward the semiconductor/dielectric interface, as N_2_ is known to have a high affinity to silicon [36]. The analysis also included oxygen and hafnium lines for all investigated samples. However, no significant differences were observed between the samples in the measured hafnium lines, i.e., Hf4f, O1s, and C1s. For the hafnium line, two components were observed, coming from the spin-orbit doublet of the Hf4f line, with the binding energy close to the table values. All identified values of the half-width and the distance of the components were consistent with the literature. The analysis of the O1s peak showed the presence of two components. The element with binding energy close to 531 eV was associated with Hf in HfO_2_ (according to the tables, 530.6 eV), while the second component came from water adsorbed on the surface, with water binding affinity levels of about 38% and 30% for optimal and non-optimal hafnium oxynitride samples, respectively. It was supposed that the nitrogen, bonded with the hydrogen, stemmed from typical impurities in the reactive chamber during the deposition process. A BE of about 403 eV corresponded well with the data published in the literature [37]. Unfortunately, only measurements at different incidence angles were possible in this work without sputtering the material bulk toward the interface. However, the angular XPS technique allowed for the measurement of the composition of the layer at various film depths, in the range of several nanometers, without sputtering. Measurements were made for the incidence angles of 0°, 30°, and 55°.

The system’s geometry indicated that, the greater the angle between the detector and the sample, the closer to the surface the data were collected. The angular measurements indicate the increased the O1s line component close to 534 eV which is related to surface contamination and is consistent with increases in the carbon content on the surface. Upon 30° and 55° geometry measurements, no increased nitrogen levels were detected on the surface of the films annealed at 800 °C. This confirmed the hypothesis that, after annealing at 800 °C, nitrogen diffused into the HfO_x_N_y_/Si interface. This hypothesis was also confirmed during the X-ray reflectometry measurements (XRR). The fitting of the experimental data to the physical model confirmed that nitrogen was located at the interface in both samples annealed at 800 °C. The changes in the nitrogen content at the HfO_x_N_y_/Si interface ensured differenced in the electrical behavior of devices fabricated employing optimal and non-optimal processes, as discussed above. It has been demonstrated in the literature that different nitrogen content could influence the performance of MOS devices—not only in the case of hafnia-based dielectric materials [38], but also in the case of SiO_x_N_y_/Si structures [39,40].

**Figure 8:**
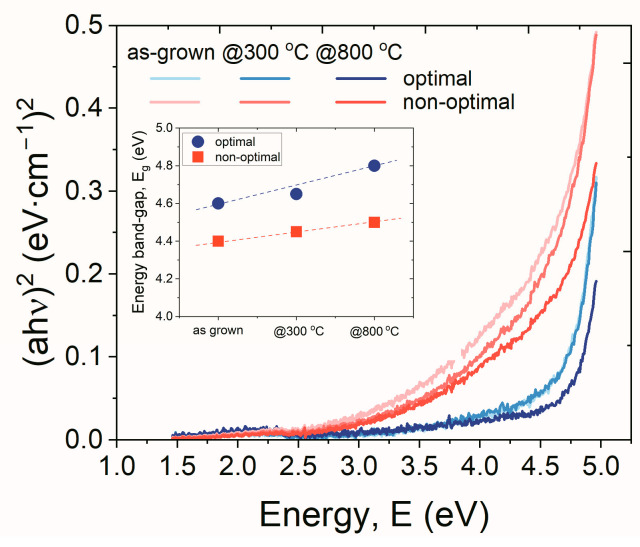
Variation of Tauc’ plots of HfO_x_N_y_ thin-films. Inset: the extracted band-gap (*E_g_*) value, based on the relation *(αhν)^2^* versus *hν* of particular hafnium oxynitride films.

The changes in the structural properties of both examined materials also influenced other parameters that characterized the dielectric properties of hafnium oxynitride. Figure 8 shows the relationship of (*αhν*)^2^ versus *hν*, determined from reflectance and transmittance measurements, and provided absorbance characteristics. The nature of optical transition was determined based on the optical absorption spectra. A plot of (*αhν*)^2^ versus *hν* would have a linear region with slope *A* and extrapolation to *α*(*hν*) = 0 would give a value of *E_g_* [41]. The spectra, as presented in Figure 8, proved that higher band-gap values characterized the HfO_x_N_y_ films deposited employing the HiPIMS optimal process, as compared to non-optimal dielectric films. The annealing temperature further increased the *E_g_* values, improving the insulating properties of the specific dielectric films discussed above. The presented *E_g_* values were consistent with results in the literature regarding band-gap identification of HfO_x_N_y_ films and lowering of the *E_g_* value with increased N content in the layer, as confirmed by XPS results [42].

**Figure 9:**
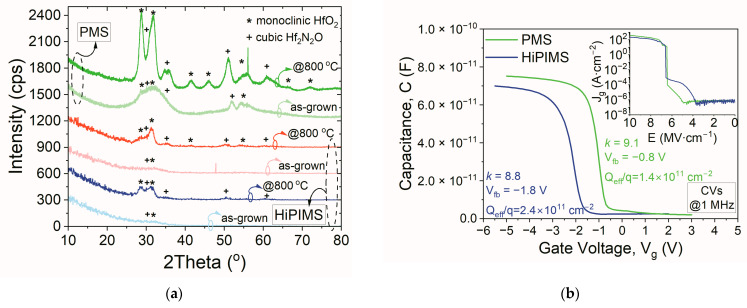
Comparison of GIXRD patterns of dielectric thin films (as-grown and annealed at 800 °C) (**a**) and C-V characteristics of MOS devices with HfO_x_N_y_ films fabricated using PMS and HiPIMS processes and annealed at 800 °C (**b**); the inset to (**b**) shows the leakage current density vs. breakdown electric field intensity *(E_br_)* of MOS structures.

In the final step of the performed study, comparisons of the electrical properties and thermal stability levels of hafnium oxynitride films fabricated by HiPIMS and typical PMS were implemented. Based on the results presented in the previous work [43], the thermal stability of the investigated dielectric films was analyzed. Figure 9a depicts the XRD data of HfO_x_N_y_ layers fabricated by HiPIMS and PMS. For the sake of easier comparison, only spectra of as-grown films and those annealed at 800 °C were shown. It was reasonable to conclude that both hafnia-based films were similar in terms of structural composition, as typical peaks, consistent with the monoclinic and orthorhombic crystalline phases, were visible. However, the intensity and number of identified peaks were significantly more prominent for materials fabricated employing the PMS procedure. Thus, the hafnium oxynitride thin films fabricated by the HiPIMS method exhibited higher crystallization temperatures and thermal stability levels than PMS HfO_x_N_y_ layers.

Moreover, the comparison of electrical behavior, as shown in Figure 9b, confirmed similar electrical parameters of the fabricated HiPIMS materials, as compared to dielectric films formed by the PMS process. It should be mentioned that the thicknesses of both dielectric film types were approximately 30 nm. Findings demonstrated that MOS structures with HiPIMS HfO_x_N_y_ were characterized by an effective charge density of 2.4 × 10^11^ cm^−2^ and a critical electric field intensity that resulted in dielectric breakdown at 6.5 MV/cm. The permittivity of the HiPIMS hafnium oxynitride was 8.8. These results were comparable to MOS structures with PMS layers. Thus, the HiPIMS process was found to be promising for further optimization of the electrical performances of deposited dielectric films.

After analysis of the obtained results, we must highlight the fact that the deterioration of electrical parameters of high-k materials, demonstrated in the literature, was only observed in our work in the deliberately non-optimal HiPIMS process. This effect was caused by a significant increase of the crystalline phase due to the thermal treatment, as also observed in [44]. In the literature, it is easy to find thermally stable high-k films, e.g., ALD HfO_x_N_y_ [45] or HfSiON [11], that have high-quality electrical properties connected with the stability of the amorphous phase of dielectric bulk. However, the findings presented in this study clearly demonstrated that the annealed HiPIMS hafnium oxynitride could be characterized by its amorphous nature, with the inclusion of a crystalline phase. Nonetheless, the electrical properties were still improved in comparison to as-grown samples. This could possibly have been due to the rebuilding of the structure of hafnium oxynitride films, improved crystallization temperatures, and the suppression of interfacial layer growth due to the presence of Si-N bonds at the dielectric/semiconductor interface [46]. Moreover, applying the orthogonal tables, according to the DoE methodology, allowed for the design and fabrication of MOS structures with HfO_x_N_y_ layers formed by the HiPIMS process with improved electrical parameters. Comparing the electrical and structural properties of hafnium oxynitride layers, fabricated by HiPIMS and PMS methods, it was reasonable to conclude that the dielectric films obtained using high-energetic pulses were denser, more electrochemically stable, and more immune to high-temperature treatment that those obtained using the typical PMS procedure.

## 4. Conclusions

The aim of this work was the development of a method by which to obtain thin hafnium oxynitride layers, employing the HiPIMS method, albeit with improved electrical parameters. The optimization procedure was implemented using Taguchi orthogonal tables. During the optimization process, the parameters of examined dielectric films were monitored employing optical methods (spectroscopic ellipsometry and refractometry), electrical characterization (C-V and I-V measurements of MOS structures), and structural investigations (AFM, XRD, XPS). The thermal stability levels of fabricated HfO_x_N_y_ layers up to 800 °C were also examined. The presented results demonstrated the correctness of the optimization methodology, as HfO_x_N_y_ layers fabricated using the optimal HiPIMS process were characterized by improved electrical parameters. These were revealed in the lower flat-band voltage (*V_fb_*) values, the disappearance of frequency dispersion of C-V characteristics, reduced effective charge (*Q_eff_/q*), and interface traps (*D_itmb_*) densities of examined MOS structures. It is worth underlining that the improved electrical properties could be correlated with the lower amount of nitrogen content in the layer bulk and the interface. Moreover, the results demonstrated the superior thermal stability of HfO_x_N_y_ layers up to 800 °C. No deterioration of electrical properties or surface morphology was observed, although a slight increase of the crystalline phase in the layer bulk was noted. The examinations of HfO_x_N_y_ layers revealed comparable electrical properties and greater immunity to thermal treatment in the dielectric films formed using HiPIMS, as compared to those obtained using the standard PMS technique. Considering the optimization results presented above, it was concluded that the investigated HiPIMS hafnia-based dielectric film could be a candidate for use in the creation of a gate dielectric layer compatible with self-aligned CMOS technology.

## Figures and Tables

**Table 1:** The values of the HiPIMS reactive magnetron sputtering process selected for optimizing the electrical properties of HfO_x_N_y_ films.

Pressure (mTorr) *	HiPIMS Power (W)	Oxygen (O_2_) Flow (sccm) *	Nitrogen (N_2_) Flow (sccm) *
3	300	5	5
6	500	10	10
9	700	15	15

* alternative units other than SI are used in this paper, as they are typical setting parameters in the apparatus used during experimental runs in this work, i.e., Oxford Instruments Plasma Technology System 400; furthermore, these units are commonly used in the literature with different fabrication techniques of dielectric and conductive materials.

**Table 2:** Experimental runs of the HiPIMS process designed for the verification of the electrical properties of HfO_x_N_y_ films.

Type of the Process	Pressure (mTorr)	HiPIMS Power (W)	Oxygen (O_2_) Flow (sccm)	Nitrogen (N_2_) Flow (sccm)
Optimal	2	100	20	3
Non-optimal	9	700	5	15

## Data Availability

The data presented in this study are available on request from the corresponding author.
